# Co-Regulations of *Spartina alterniflora* Invasion and Exogenous Nitrogen Loading on Soil N_2_O Efflux in Subtropical Mangrove Mesocosms

**DOI:** 10.1371/journal.pone.0146199

**Published:** 2016-01-04

**Authors:** Dai Jia, Fei Qi, Xia Xu, Jianxiang Feng, Hao Wu, Jiemin Guo, Weizhi Lu, Ronghao Peng, Xiaoshan Zhu, Yiqi Luo, Guanghui Lin

**Affiliations:** 1 Ministry of Education Key Laboratory for Earth System Modeling, Center for Earth System Science, Tsinghua University, Beijing, China; 2 Shenzhen Key Laboratory for Coastal Ocean Dynamic and Environment, Division of Ocean Sciences and Technology, Graduate School at Shenzhen, Tsinghua University, Shenzhen, China; 3 Key Laboratory of the Ministry of Education for Coastal and Wetland Ecosystems, School of Life Sciences, Xiamen University, Xiamen, China; 4 Department of Microbiology and Plant Biology, University of Oklahoma, Oklahoma City, OK, United States of America; Lakehead University, CANADA

## Abstract

Both plant invasion and nitrogen (N) enrichment should have significant impact on mangrove ecosystems in coastal regions around the world. However, how N_2_O efflux in mangrove wetlands responds to these environmental changes has not been well studied. Here, we conducted a mesocosm experiment with native mangrove species *Kandelia obovata*, invasive salt marsh species *Spartina alterniflora*, and their mixture in a simulated tide rotation system with or without nitrogen addition. In the treatments without N addition, the N_2_O effluxes were relatively low and there were no significant variations among the three vegetation types. A pulse loading of exogenous ammonium nitrogen increased N_2_O effluxes from soils but the stimulatory effect gradually diminished over time, suggesting that frequent measurements are necessary to accurately understand the behavior of N-induced response of N_2_O emissions. With the N addition, the N_2_O effluxes from the invasive *S*. *alterniflora* were lower than that from native *K*. *obovata* mesocosms. This result may be attributed to higher growth of *S*. *alterniflora* consuming most of the available nitrogen in soils, and thus inhibiting N_2_O production. We concluded that N loading significantly increased N_2_O effluxes, while the invasion of *S*. *alterniflora* reduced N_2_O effluxes response to N loading in this simulated mangrove ecosystem. Thus, both plant invasion and excessive N loading can co-regulate soil N_2_O emissions from mangrove wetlands, which should be considered when projecting future N_2_O effluxes from this type of coastal wetland.

## Introduction

Although nitrous oxide (N_2_O) contributes only 5% to estimated global warming potentials [1], its global warming strength is 265 times more powerful than CO_2_ over a 100-year time frame [2]. N_2_O has also contributed to destruction of ozone in the stratosphere[3]. During the past few decades, the global mean atmospheric concentration of N_2_O increased from 270 ppb in pre-industrial times to 324 ppb in 2011[2]. The rapid increase in atmospheric N_2_O concentrations has gained much attention in quantifying N_2_O effluxes from various sources.

Coastal wetlands, such as mangroves in tropical and subtropical regions have been recognized as a major marine source of atmospheric N_2_O [4, 5]. In general, N_2_O effluxes from wetlands are related to several biological processes, mainly including nitrification (ammonium oxidation and nitrifier denitrification) [6], denitrification and nitrate-ammonification [7]. These processes can be affected by many abiotic and biotic factors, such as soil temperature, oxygen level, and substrate availability (bioavailable carbon, ammonium and nitrate) in the soil [8, 9]. In addition, it is widely recognized that vegetation type can significantly affect N_2_O effluxes [10]. Plants living in wetland environments can supply oxygen to the rhizosphere via aerenchymous tissue of plants, which creates oxidized microzones surrounding roots and rhizomes and favors nitrification and nitrification-denitrification reactions, facilitating N_2_O production [11–15]. Meanwhile, root exudates and debris can be used as an organic carbon source for microbes. The high activity of microbes could increase soil N mineralization [16] and promote N_2_O production. Therefore, the change in vegetation type, such as plant invasion, may have a potential effect on N_2_O effluxes.

*Spartina alterniflora*, a C_4_ salt marsh grass native to the east coast of the USA, was introduced to China in 1979. It has grown vigorously in China and spread over coastal wetlands since that time. During the last two decades, it has aggressively invaded habitats of native mangroves on the southern coast of China, especially in mangrove seedlings established areas [17–19], influencing the native species composition of mangrove ecosystems. As a result, it would alter soil available inorganic nitrogen (N) content and microbial activity [20, 21], which would further change N_2_O production and effluxes. So, it is essential to understand the change of N_2_O effluxes from mangrove wetlands following *S*. *alterniflora* invasion.

Globally, many mangrove ecosystems are also under significant threat from N pollution because of human activities, such as sewage discharge and surrounding aquaculture operation [22]. Exogenous N loading has greatly changed functions of many mangrove wetlands in tropical and subtropical regions, from pristinely oligotrophic [23] to eutrophic [24]. Usually, pristine mangrove soils, in N-limited environments, show low or negative N_2_O effluxes [25, 26]. Exogenous N loading often stimulate N_2_O effluxes by directly stimulating nitrification and denitrification in the soil or sediment [27, 28]. For example, the Futian mangrove swamp in China, an area with intense human activities, had the highest N_2_O effluxes compared to three other mangrove swamps with slightly anthropogenic disturbance [27]. Similarly, the effluxes of N_2_O from the Puerto Rico mangrove swamp in the northeastern Caribbean Sea, where anthropogenic activities increase nitrogen loading to mangrove sediments, was much higher than those reported previously for intertidal estuarine sediments [29].

In the coastal wetlands, N loading from surrounding aquaculture ponds are always periodic. For example, during the common process of complete pond sediment cleaning, large quantities of sediment with high nutrient levels are washed out and discharged into the adjacent mangrove swamps in a very short period of time [30]. N_2_O effluxes may vary greatly over time because of dynamic changes of soil inorganic N concentration after such exogenous N loading. In order to accurately estimate N loading effect on N_2_O effluxes, attention should be paid to dynamic changes of N_2_O effluxes after a pulse of exogenous N loading. However, to our knowledge, there has been limited research focusing on the dynamic change of N_2_O effluxes after an N-pulse loading in a wetland (but see [31]).

Although the previous research has pointed out that N_2_O effluxes in mangroves have positive response to excessive N loading, the response ability would be different between vegetation types [32]. After N loading, the divergent growth rate between various species meant dissimilar uptake capabilities of ammonium nitrogen (NH_4_^+^-N) and nitrate nitrogen (NO_3_^-^-N), which are the important substrates participating in nitrification and denitrification processes. The difference in the competition between microorganisms and plants for N may lead to a differential in soil N_2_O production [33, 34]. Thus, to gain an insight into the relationship between excessive N loading and N_2_O effluxes from mangrove wetlands after *S*. *alterniflora* invasion, the varying physiological responses of various plant species should also be considered.

In order to understand individual and possible interactive effects of *S*. *alterniflora* invasion and exogenous N loading on N_2_O effluxes from mangrove ecosystems, we conducted a mesocosm experiment with three vegetation types including the monocultures of *K*. *obovata* (a common native mangrove species in China) and *S*. *alterniflora* and their mixture and two N loading treatments (with or without N loading). The aims of the study were (1) to determine the dynamic change of N_2_O effluxes after a pulse N loading, (2) to test whether and how the invasion of *S*. *alterniflora* alters N_2_O effluxes, and (3) to investigate the interactive effect of plant invasion and N loading on mangrove N_2_O effluxes. We hypothesized that N loading will increase N_2_O effluxes from both mangrove and salt marsh mesocosms, but the invasive *S*. *alterniflora* mesocosms would have a lower increment of N_2_O effluxes after N loading due to larger N uptake compared with the native mangrove mesocoms.

## Materials and Methods

### Experimental design

**Fig 1:**
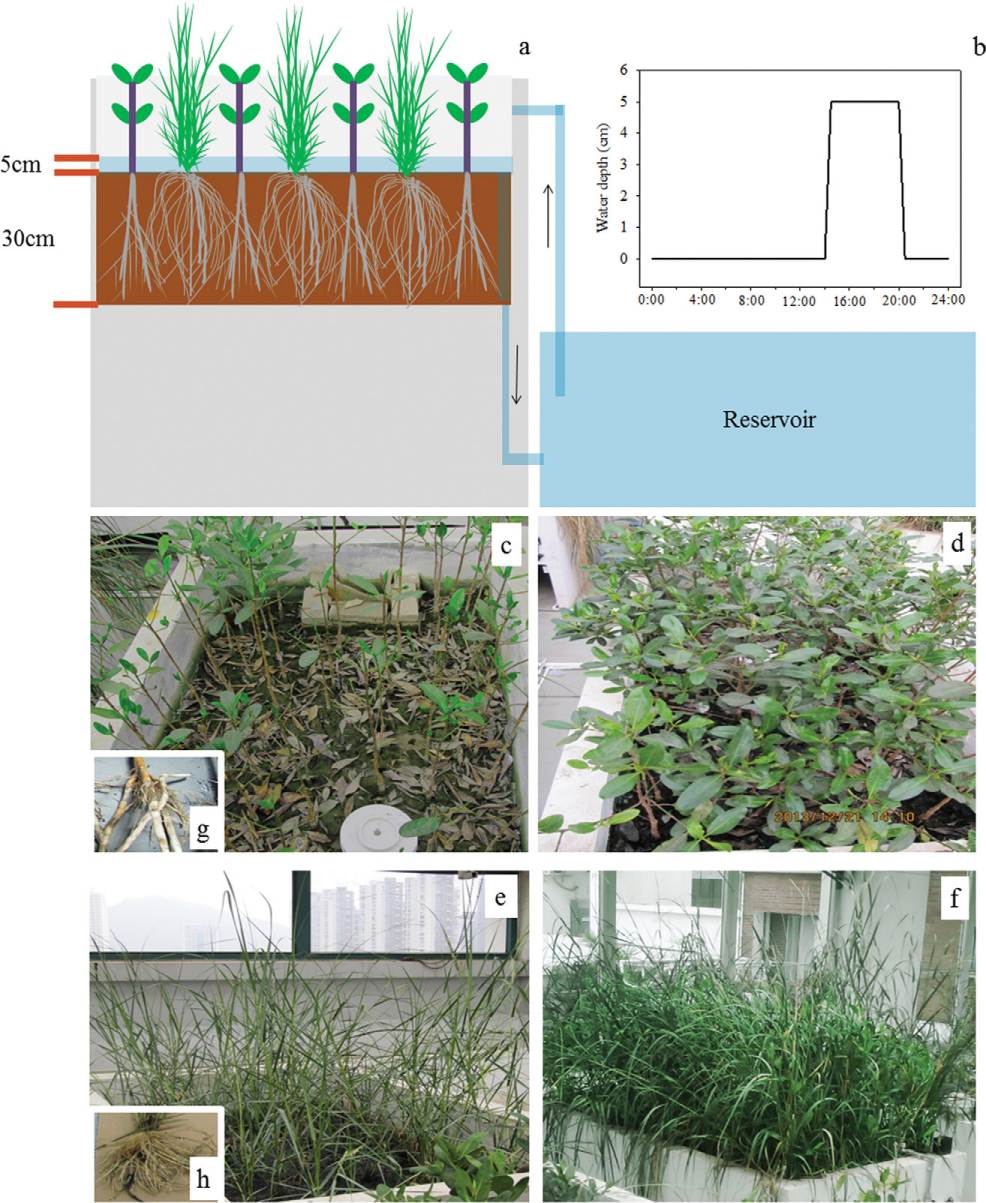
Section view of the experimental mesocosms used in the experiment (a) and the changes of water depth in the experimental mesocosms with time (b). See Section 2.1 for details; the picture of mesocosms (c: *K. obovata* (KO) without N addition, d: KO with N addition, e: *S. alterniflora* (SA) without N addition, f: SA with N addition); the root of KO (g) and SA (h).

The tide-system mesocosm experiment was conducted in a roof greenhouse at the Graduate School at Shenzhen, Tsinghua University, China (22°59′N, 113°97′E). The mesocosm system consisted of 18 cement tanks (2.40 m × 1.10 m × 0.50 m in volume) as experimental mesocosms and 2 cement tanks (7.50 m × 1.00 m × 0.70 m in volume) as seawater reservoirs (Fig 1). One reservoir was connected to all the mesocosms without N addition treatment and the other was connected to all the mesocosms with N addition treatment by pipelines of water inputting (used during tide flooding periods) and water outputting (used in tide falling periods). The artificial seawater at a salinity of 14 g l^-1^, representing an average salinity in a typical mangrove environment of southern China, was prepared by dissolving natural sea salts in tap water. Submersible pumps and timers were installed to simulate diurnal tides for all the mesocosms. For the transplanted plants to establish and acclimate, each mesocosm was conditioned under the tidal regime with one tidal cycle a day, 12 h high tide and 12 h low tide for 2 months before starting the experiment on August 31, 2012. After the formal start of the system operation, artificial seawater was pumped from the reservoir to each mesocosm to a depth of 5 cm between 14:00 and 20:00 (local time) to simulate high tide, then seawater was drained back by gravitational force to the reservoir during the low tide period. Tidal flux took about 30 minutes from the initiation of the ebb or flood tide. Tidal water in each reservoir was rotated for about 15 days as a water cycle, then drained and replenished by freshly prepared artificial seawater. In this way, we mimicked field conditions because daily tidal dynamic change might greatly change nitrification and denitrification potential, allowing us to determine whether the effects were robust with respect to natural perturbations.

Each mesocosm was filled with fresh mangrove soils. On June 5, 2012, the mangrove soils were collected from the mudflat connected to the Futian Mangrove Natural Reserve (22°51'N, 113°96'E), a typical mangrove swamp along the southern coast of Shenzhen. We collected the soils after receiving the permission of the Futian Mangrove Nature Reserve in this research. The substrate was sandy, consisting of 73.7% sand, 14.8% silt and 11.6% clay. After stones, benthic animals and plant residues in the soil were carefully removed, the soils was then used to fill each mesocosm to a depth of 30 cm (Fig 1). In the present study, *K*. *obovata* was chosen because it was the most dominant species among the eight true mangrove species in South China [35]. The mature propagules of *K*. *obovata* and ramets of *S*. *alterniflora* were collected from the mangrove forest in Zhangjiangkou Mangrove Nature Reserve in Yunxiao, Fujian (23°56'N, 117°25'E). We collected the plants after receiving the permission of the Zhanjiang Mangrove National Nature Reserve in this research. The propagules of *K*. *obovata* were cultivated in the sand for 2 months before transplantation to the mesocosms. Then healthy *K*. *obovata* seedlings with approximately equal height of 15 cm (usually with 3–5 green leaves) were selected for this study. Meanwhile, the young ramets of *S*. *alterniflora* of approximately equal size with four true leaves were selected for this study. Each *K*. *obovata* seedling and ramet of *S*. *alterniflora* covered an area of 0.15 m × 0.15 m and 0.25 m × 0.25 m, respectively. This mimics typical seedling densities of these two species according to our observations in the field.

**Fig 2:**
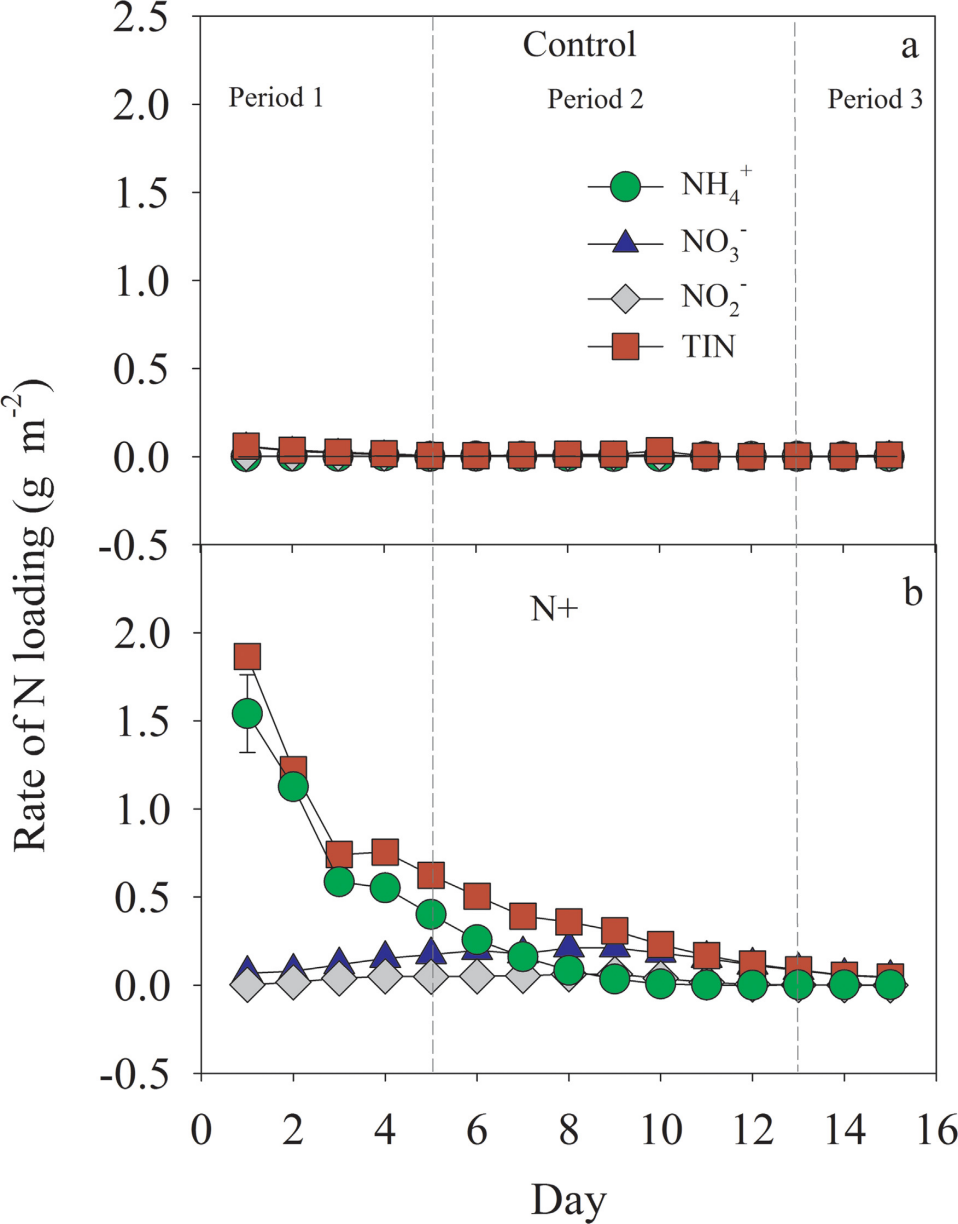
The dynamics of rates of ammonium nitrogen (NH_4_^+^-N), nitrate nitrogen (NO_3_^-^-N), nitrite nitrogen (NO_2_^-^-N) and total inorganic nitrogen (TIN-N) loading with (b) and without (Control: a) N addition during the whole experiment period.

In this experiment, three vegetation types represented three stages of *S*. *alterniflora* invasion to *K*. *obovata*: (i) monoculture of *K*. *obovata* (no invasion), (ii) mixture of *K*. *obovata* and *S*. *alterniflora* (partial invasion), and (iii) monoculture of *S*. *alterniflora* (complete invasion) (Fig 1). All vegetation types were tested in the absence and presence of nitrogen addition. Ammonium chloride (NH_4_Cl) of 450 mg was added to synthetic seawater of 3000 ml in the reservoir connected to mesocosms with N addition treatment one day before the start of water cycle (i.e. one day before tide rising of the first day) in order to make NH_4_Cl dissolve thoroughly. During a typical 15 day water cycle period, no more NH_4_Cl was added into the reservoir. Because the N of water may be lost by transformation by microbes and volatilization in form of ammonia gas in the reservoir during low tide periods, we measured the actual rates of N loading, which were shown in Fig 2. The N levels we applied are within the range of sewage and wastewater and sludge from aquaculture ponds [36, 37].

### Measurements of N_2_O effluxes

For the results reported in this paper, we selected a typical 15 day water cycle period. Artificial seawater pumping started at 14:00–20:00 on June 18, 2013 (Day 1) from a new pool of tidal water with desired salinities and N levels specified above. We started the collection of gas samples on the day after a new seawater change (i.e. on June 19, 2013) to investigate the effect of N loading on N_2_O effluxes during daytime of tide falling periods. During this water cycle period, the N_2_O effluxes in each mesocosm were quantified using a static chamber-gas chromatograph method on the day 2, 4, 6, 8, 10, 14 and 16. Each static chamber, which was made of PVC, covered a soil area of 0.025 m^2^ and had an internal volume of about 4000 ml. The open end of the chamber was inserted 3–5 cm into the soil with the air-tight valve open. Gas sampling was carried out 30 minutes after the chamber was inserted. Generally, five 8 ml gas samples of chamber air were collected by passing a hypodermic needle attached to a 10 ml glass syringe and was injected into pre-evacuated vials for laboratory analysis. Meanwhile, to minimize any effect of diurnal variation in N_2_O effluxes, samplings were conducted at roughly the same time of the day for the same mesocosm between 9:00 and 11:00. The deployment time was set to 45–90 min with sampling at 15–30 min intervals depending on the N_2_O efflux rates.

The N_2_O concentration of gas samples collected was analyzed within 24 h after sampling using a gas chromatograph (GC, Agilent 6820, Agilent Technologies, USA) equipped with an electron capture detector (mECD) and a HP- Plot/column (J&W GC Columns, Agilent Technologies, USA). The temperatures of the injector, column and detector were 50°C, 50°C and 300°C, respectively, with a carrier gas (N_2_) flow rate of 15 ml min^-1^. The relative standard deviation (RSD) of replicate standard measurements was 0.8%. During gas measurements, standard samples were analyzed with every 10 samples of determination to ensure that each sample run maintained a RSD below 3% in 12 h. N_2_O concentrations were quantified by comparing the peak areas of samples against the standard curves. N_2_O efflux rates were calculated from the linear change of the measured gas concentrations in the chamber headspace with known headspace volume and sampling time. Estimates of cumulative N_2_O emissions for each mesocosm were based on linear interpolation, with the sum of cumulative experiment period emissions representing one water cycle.

### Sampling and analyses of water and soil

Everyday input water samples from each reservoir were collected before the start of tide flooding and output water samples from each mesocosm were collected before the start of tide falling during the experiment water cycle period. The water samples were transported to the laboratory and analyzed immediately. The samples were filtered through a 0.45 μm filter and analyzed for NH_4_^+^-N, NO_3_^-^-N, nitrite nitrogen (NO_2_^-^-N). We used standard procedures and methods for the determination, including the Sodium salicylate-sodium hypochlorite Method for NH_4_^+^-N, the UV Spectrophotometer Method for NO_3_^-^-N and the α-Naphthylamine Method for NO_2_^-^-N. Total inorganic nitrogen (TIN-N) was calculated by summing NH_4_^+^-N, NO_3_^-^-N and NO_2_^-^-N [38]. Rates of N loading were calculated by multiplying N concentration of input water by the area and the flooding depth (5cm) of each mesocosm.

Three soil cores (0–15 cm) were collected around the chamber using hand-held PVC corers from each mesocosm at day 4 after gas sampling. Soil total carbon and nitrogen contents were measured by the Elementar (Vario ELⅢ, German). The NH_4_^+^-N and NO_3_^-^-N contents in the KCl (2 M) extracts were also determined by the methods above. Both soil nitrification potential activity (PNA) and denitrification potential activity (PDA) were measured according to the method of Chen et al. (2012) [39].

### Measurements of plant biomass

*K*. *obovata* biomass, measured on July 5, 2013, was evaluated by a non-destructive allometric technique [40]. The relationships between biomass (dry weight) of each plant part, namely leaf, stem and plumular axis, and their leaf number (LN), and stem height (SH), propagule height (PH) and basal diameter (D) were obtained by harvesting 12 individuals of *K*. *obovata* from each mesocosm randomly. The best-fit equations for estimating biomass of leaves (LB), stems (SB) and propagules (PB) were LB = 0.32 LN − 0.89 (*r*^*2*^ = 0.89), SB = 0.23 × SH × D × D + 0.40 (*r*^*2*^ = 0.80), and PB = 0.52 × PH × D × D + 2.53 (*r*^*2*^ = 0.85), respectively. The total aboveground biomass was calculated by summing LB, SB and PB. The best-fit equations for estimating the total belowground biomass was BGB = 0.31 × AGB +1.16 (*r*^*2*^ = 0.85).

*S*. *alterniflora* biomass was measured also on July 5, 2013 but was evaluated by a non-destructive height–weight method [41]. The relationships between aboveground biomass (B) of plant and their height (H) were obtained by harvesting 200 individuals of *S*. *alterniflora* from each mesocosm randomly. The best-fit equations for estimating aboveground biomass of was ln B = 1.44 ln H − 5.46 (*r*^*2*^ = 0.85).The best-fit equations for estimating BGB of were ln BGB = 0.61 × AGB + 34.88 (*r*^*2*^ = 0.86).

Plant samples were analyzed for N concentration with a Elementar elemental analyzer (Vario ELⅢ, German) and the total N accumulation was calculated by multiplying the N concentration by dry weight.

### Statistical analyses

The main and interactive effects of sampling time, vegetation type and nitrogen addition treatment on N_2_O effluxes were tested with a parametric three-way analysis of variance (ANOVA). A one-way analysis of variance (ANOVA) performed to examine the significant differences of N_2_O effluxes between vegetation types for each period. One-way ANOVA was also used to analyze the differences in parameters of soil properties and plant traits between the treatments, followed by the least significant difference (Duncan) test at *P* < 0.05. Linear regression analyses were performed to evaluate the relationships of NH_4_^+^-N, NO_3_^-^-N, NO_2_^-^-N and TIN-N loading rates and N_2_O effluxes. The student's t-test was used to examine the significant difference of slopes and intercepts of linear regression lines of TIN-N and N_2_O effluxes, and NH_4_^+^-N and N_2_O effluxes, NO_3_^-^-N and N_2_O effluxes and NO_2_^-^-N and N_2_O effluxes. All statistical analyses were performed using SPSS 19.0 for Windows (SPSS Inc. USA).

## Results

### Rate of N loading

In the treatment without N addition, NO_3_^-^-N was the dominant dissolved N form of the input water and contributed 70.08% of TIN-N load. The rate of NO_3_^-^-N loading declined gradually with time. With N addition, after the refreshment of tidal water, the rate of NH_4_^+^-N loading in the mesocosms decreased with time from 1.54 to 0.002 g m^-2^, However, the rate of NO_3_^-^-N loading in the mesocosms increased to a peak by the day 8 (0.21 mg m^-2^), and then decreased gradually to 0.04 mg m^-2^. A similar variation pattern was observed for nitrite (NO_2_^-^-N), with the highest rate (0.06 mg m^-2^) also on day 8. The rate of TIN-N loading generally decreased with time (Fig 2).

### Soil properties and plant traits

In treatments without N addition, concentrations of soil total carbon, nitrogen, NH_4_^+^-N and NO_3_^-^-N were not significantly affected by plant community composition, except that the NH_4_^+^-N concentration in mixture mesocosms was significantly lower than that in *S*. *alterniflora* mesocosms and *K*. *obovata* mesocosms. In treatments with N addition, NH_4_^+^-N concentrations differed among the three plant communities, ranging from 2.25 mg kg^-1^ in the *S*. *alterniflora* mesocosms, and 3.02 mg kg^-1^ in the mixture mesocosms to 3.27 mg kg^-1^ in the *K*. *obovata* mesocosms. PNA also varied with plant species. N loading increased the soil total carbon, NH_4_^+^-N and PNA significantly. However, soil total N, NO_3_^-^-N and PDA were not significantly different between the N addition and without N addition mesocosms (Table 1).

**Table 1:** Soil and plant properties for the *K*. *obovata* (KO), *S*. *alterniflora* (SA) and their mixture mesocosms with and without (Control) exogenous N addition.

	Control			N addition		
	KO	Mix	SA	KO	Mix	SA
**Soil total carbon (g C kg**^**−1**^**)**	8.58±1.18ab	7.15±0.47a	7.51±0.34a	8.77±0.77b	8.20±0.21b	8.81±1.11b
**Soil total nitrogen(g N kg**^**−1**^**)**	0.33±0.01a	0.30±0.03a	0.32±0.02a	0.30±0.02a	0.34±0.02a	0.32±0.02a
**NH_4_^+^-N(mg N kg^-1^)**	1.83±0.62bc	0.55±0.03a	1.06±0.15b	3.27±0.29d	3.02±0.97cd	2.25±0.36c
**NO_3_^-^-N (mg N kg^-1^)**	1.72±0.27a	1.72±0.16a	1.8±0.28a	2.45±0.42a	1.77±0.30a	2.38±0.44a
**PNA (mg NO_2_^-^-N g^-1^ DW d^-1^)**	0.34±0.01a	0.49±0.01b	0.39±0.01a	1.89±0.02d	1.61±0.08d	0.83±0.04c
**PDA (μmol N**_**2**_**O-N g**^**-1**^ **DW d**^**-1**^**)**	0.11±0.01a	0.19±0.04a	0.16±0.02a	0.11±0.01a	0.13±0.01a	0.14±0.01a
**Water content (%)**	0.52±0.02a	0.47±0.03a	0.52±0.10a	0.51±0.06a	0.46±0.03a	0.47±0.05a
**Initial AGB (kg m**^**-2**^**)**	0.22±0.003c	0.14±0.007b	0.09±0.005a	0.22±0.003c	0.15±0.006b	0.08±0.007a
**AGB (kg m**^**-2**^**)**	0.58±0.06a	0.65±0.04a	0.62±0.09a	0.90±0.13b	1.92±0.19c	2.03±0.19c
**Initial BGB (kg m**^**-2**^**)**	0.07±0.001b	0.09±0.003a	0.09±0.006a	0.07±0.001c	0.1±0.007a	0.08±0.006a
**BGB (kg m**^**-2**^**)**	0.18±0.02a	0.77±0.11c	1.02±0.18c	0.28±0.04b	2.01±0.82d	2.05±0.07d
**Total N accumulation of plant (g m**^**-2**^**)**	6.29±0.85a	14.94±0.48b	21.91±3.41c	11.69±2.24b	45.8±5.69d	53.65±8.28d

Different letters within the same row indicate significant difference at *P* < 0.05. AGB: aboveground biomass; BGB: belowground biomass; Initial AGB and BGB: AGB and BGB measured at the 1st water cycle in September, 2012; PNA: nitrification potential activity; PDA: denitrification potential activity.

Without N addition, no significant differences in aboveground biomass were found among vegetation types, while the belowground biomass in *S*. *alterniflora* mesocosms was 1.02 kg m^-2^ significantly higher than that in *K*. *obovata* mesocosms. N addition increased total biomass in the *S*. *alterniflora*, *K*. *obovata* and their mixture mesocosms by 55.26%, 176.76% and 148.78%, respectively. Under N addition condition, above- and below-ground biomass of *S*. *alterniflora* were evidently higher than that of *K*. *obovata* mesocosms. However, the ratio of aboveground to belowground biomass was 3.21 in *K*. *obovata* mesocosms, significantly higher than 0.99 in *S*. *alterniflora* mesocosms. Therefore, *S*. *alterniflora* was more productive compared to *K*. *obovata* (Table 1).

### N_2_O effluxes

**Fig 3:**
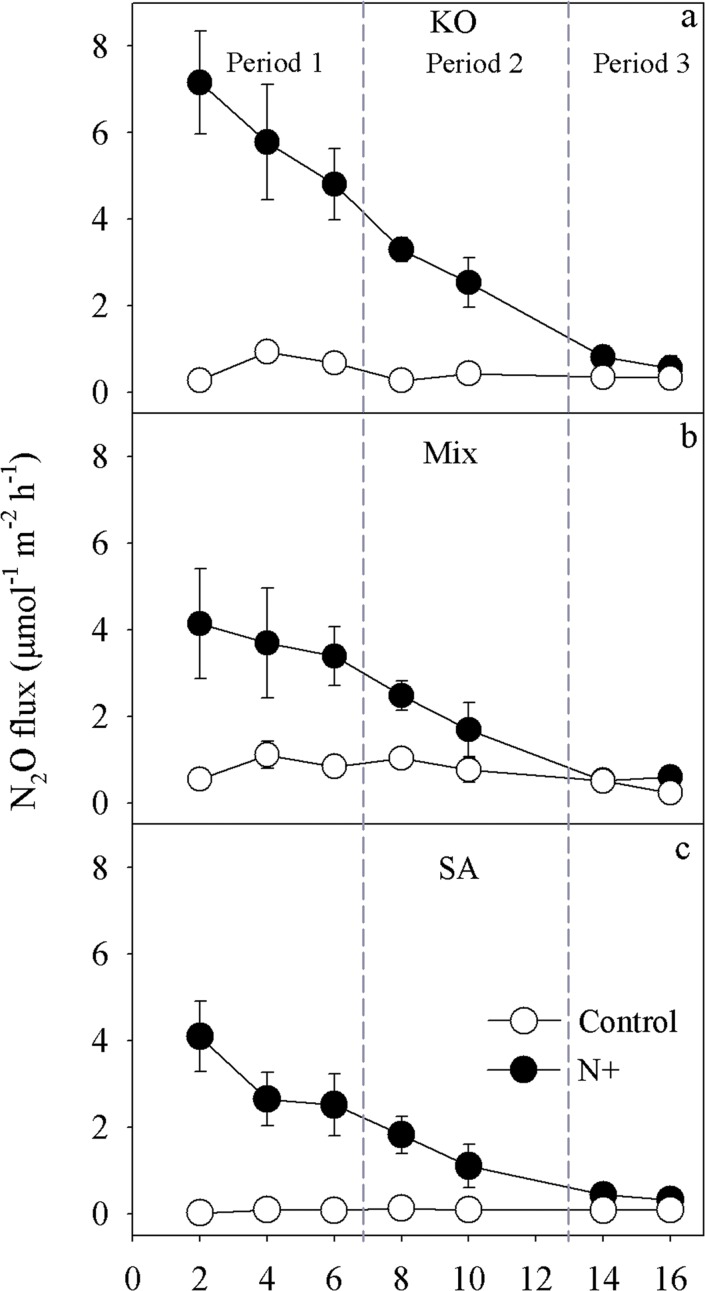
Variation of N_2_O effluxes in different vegetation types (a: KO, b: mixture and c: SA) with and without N addition (Control) during the entire experiment period. See Fig 1 for abbreviations.

Without N addition, the N_2_O effluxes from *K*. *obovata*, the mixture and the *S*. *alterniflora* mesocosms ranged from 0.27 to 0.93, from 0.52 to 1.11 and from 0.32 to 0.70 μmol m^-2^ h^-1^ in the, respectively (Fig 3). N addition significantly increased mean N_2_O effluxes (Table 2). With N addition, the N_2_O effluxes ranged from 0.56 to 7.17, from 0.23 to 4.14 and from 0.19 to 4.10 μmol m^-2^ h^-1^ in the *K*. *obovata*, the mixture and the *S*. *alterniflora* mesocosms, respectively (Fig 3). N_2_O effluxes for all three vegetation types generally decreased with sampling time. In addition, there was a significant interaction of plant species × N fertilization on N_2_O effluxes (Table 2).

**Table 2:** Results of three-way ANOVA on the effects of vegetation type (V), sampling time (individual day, D), N addition treatment (N) and their interactions on N_2_O effluxes during the entire experimental period, and repeated measures ANOVA on the effects of V, N and their interactions on N_2_O effluxes for the three periods.

		df	*P*
	**V**	2	<0.001
	**N**	1	<0.001
	**D**	6	<0.001
**Whole experiment period**	**V × N**	2	<0.001
	**V × D**	12	0.393
	**N × D**	6	<0.001
	**V × N ×D**	12	0.841
	**V**	2	0.003
**Period 1 (Day 1–6)**	**N**	1	<0.001
	**V× N**	2	0.003
	**V**	2	0.019
**Period 2 (Day 7–13)**	**N**	1	<0.001
	**V× N**	2	0.01
	**V**	2	0.227
**Period 3 (Day 14–16)**	**N**	1	0.709
	**V× N**	2	0.27

df: degree of freedom

**Fig 4:**
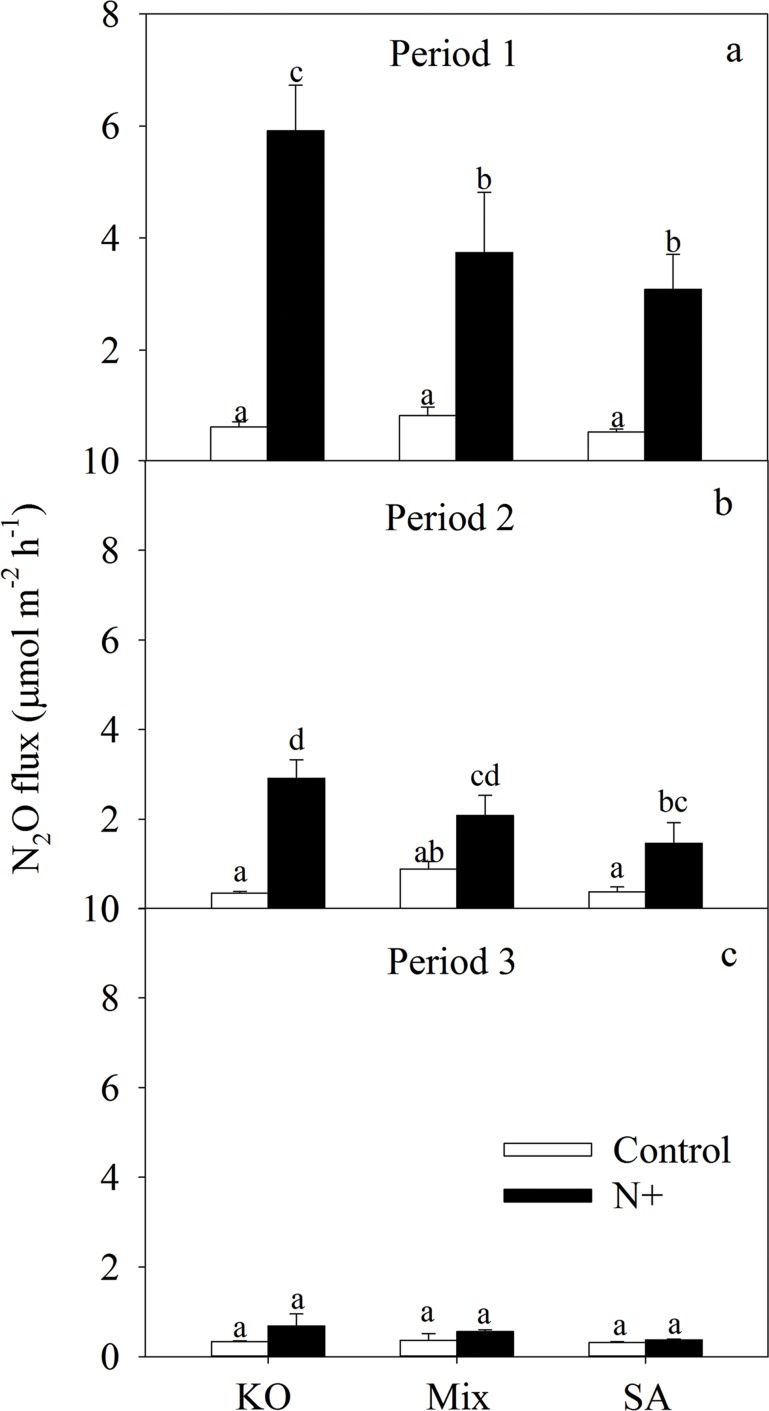
Mean rates of N_2_O effluxes from mesocosms with different vegetation types (a: KO, b: mix and c: SA) with and without N addition (Control) for the three periods. See Fig 1 for abbreviations.

We further analyzed N_2_O effluxes over the 15-day period by dividing the sampling time into three periods according to the N loading rate and main species of N in the input water with N enrichment as shown in Fig 3. NH_4_^+^-N was the major species of N in the first period during day 1 to 6 with high N loading (TIN-N loading rates ranging from 0.51 to 1.87 g m^-2^); NO_3_^-^-N was the major species of N in the second period during day 7 to day 13 with modest N loading (TIN-N loading rates ranging from 0.12 to 0.39 g m^-2^) and in the last period total N loading rates have decreased to background level during day 14 to day 16 (TIN-N loading rates ranging from 0.047 to 0.057 g m^-2^) (Fig 2).Without N addition, the N_2_O effluxes from *K*. *obovata*, the mixture and the *S*. *alterniflora* mesocosms did not vary significantly between vegetation types during all three periods, while there were trends that N_2_O effluxes were higher from mixture mesocosms than that from *K*. *obovata* and *S*. *alterniflora* mesocosms during all three periods. With N addition, compared to that from *K*. *obovata* mesocosms, the mean N_2_O effluxes were significantly lower from the *S*. *alterniflora* mesocosms in period 1 and 2 (Fig 4). However, in period 3, there were no significant difference among the vegetation types.

### Relationship between N_2_O effluxes and N loading

**Fig 5:**
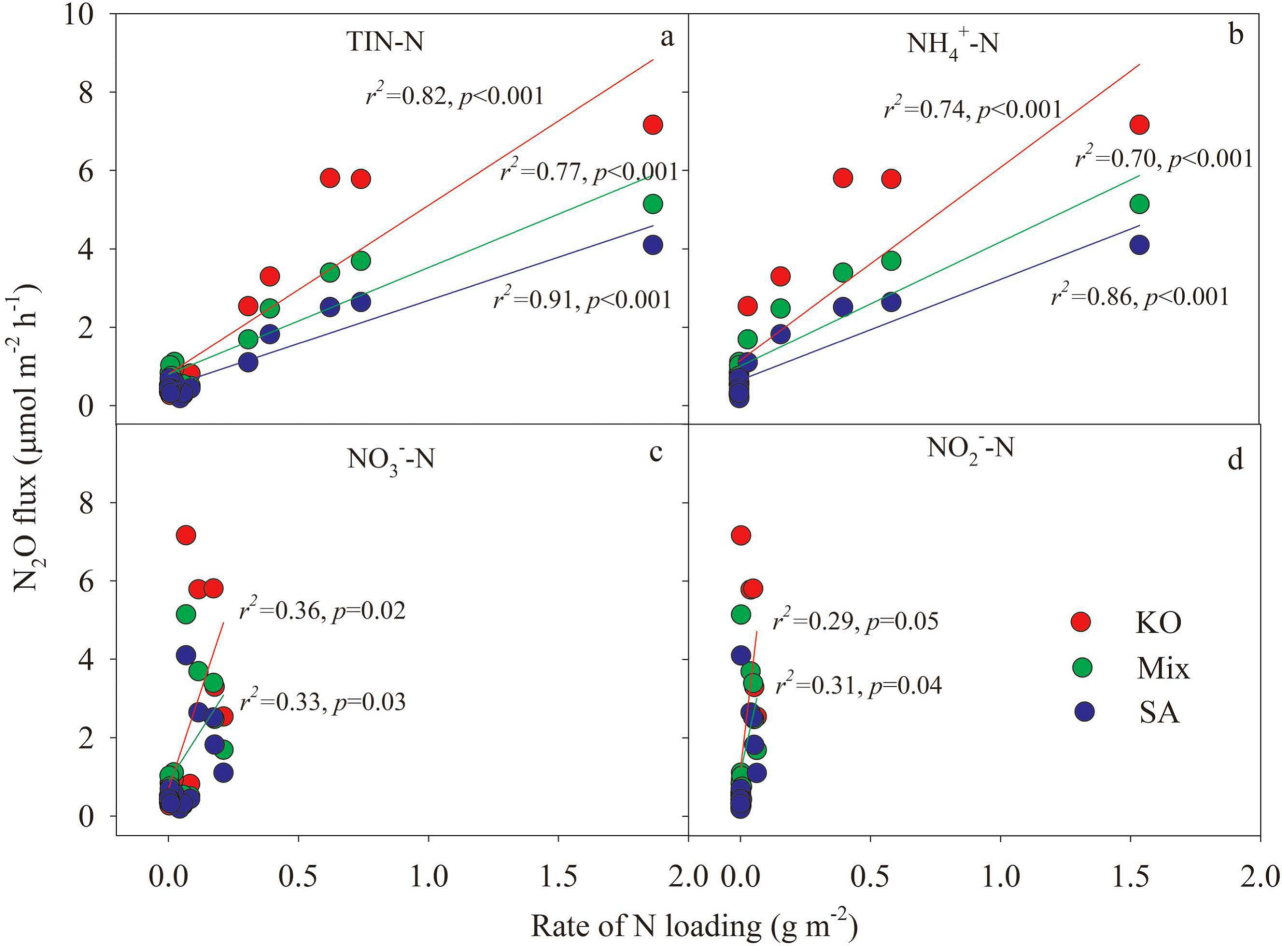
Relationships between N_2_O effluxes and rate of TIN-N (a), NH_4_^+^-N (b), NO_3_^-^-N (c) and NO_2_^-^-N (d) loading for different vegetation types (n = 14).

When integrating all data either with or without N addition for analysis in each vegetation type respectively, N_2_O effluxes were found to significantly correlate to the rate of TIN-N, NH_4_^+^-N, NO_3_^-^-N and NO_2_^-^-N loading (*P* < 0.05), except for the relationship between N_2_O effluxes and NO_3_^-^-N & NO_2_^-^-N loading rate in the *S*. *alterniflora* mesocosms. The slopes of the linear relationship between rate of TIN-N loading and N_2_O effluxes for *S*. *alterniflora* and mixture mesocosms were significantly lower than that for *K*. *obovata* mesocosms by student's t-test (*P* < 0.05) (Fig 5). Thus, the increase rate of N_2_O effluxes by N addition was much lower in the *S*. *alterniflora* and mixture than native *K*. *obovata* mesocosms.

## Discussion

Our results from this mesocosm experimental study showed that a pulse loading of exogenous ammonium nitrogen resulted in a stimulatory effect on N_2_O effluxes but the effect gradually diminished over time and that the invasion of *S*. *alterniflora* could mitigate N_2_O effluxes following N loading. In addition, there was a significant antagonism effect between plant invasion and exogenous N loading on mangrove N_2_O effluxes. Our findings offer new insights into how plant invasion and N loading modulate N_2_O effluxes from mangrove wetlands.

### Pulse effect of N addition on N_2_O effluxes

Compared to the treatment with N addition, we found that the N_2_O effluxes were relatively low (<1.50 μmol m^-2^ h^-1^) in the treatment without N addition in the *K*. *obovata*, *S*. *alterniflora* and their mixture mesocosms, which was consistent with the conclusions from the previous studies that natural coastal wetlands were weak sources for atmospheric N_2_O, such as undisturbed mangrove and salt marshes ecosystems [26, 27]. However, we did not observe any N_2_O sink in either pure *S*. *alterniflora* stand or the mixture of *S*. *alterniflora* and native mangrove seedlings, which was different from the results of Yuan, et al. (2014) [26]. A likely explanation for this difference is that in our study soil nitrate levels never reached the very low level (< 1 mg N kg^-1^) [42].

N_2_O effluxes were significantly increased by the N loading in both mangrove and *Spartina* mesocosms, which was also found in salt marshes [34]. Although N_2_O emissions accounted for a very low proportion of total N loading, ranging from 0.23–0.47% for the mesocosms with N addition treatment and from 2.22 to 3.55% for those without N loading (S1 Table), we could not neglect N loading effect on N_2_O emission, considering 265 times higher potential warming strength than CO_2_. Previous research also found relative lower proportions (ranging from 0.05–0.48% at N loading rate of 1.4 g m^-2^ in field experiment of salt marsh coastal wetland) [31]. The stimulating effect of N loading on N_2_O effluxes results might be attributed to the fact that enzyme activities and the microbial population sizes including nitrifiers and denitrifiers in surface sediment had a dramatic increase after N addition [43, 44] and N loading supply the source of substrate for nitrification and denitrification in the soil or sediment. However, for the exgenous N loading, N_2_O effluxes may be underestimated or overestimated if they were not measured continuously after the N loading, as shown in this study that the stimulatory effect of N loading on N_2_O effluxes decreased gradually over time. A contributing factor to this result is that N_2_O fluxes were significantly related to N loading rates and these N loading rates usually decreased gradually after a pulse of nitrogen loading due partly to assimilation by wetland plants, transformation by microbes, and adsorption to sediments. So, N addition significantly increased the mean N_2_O effluxes in the first (Day 0–5) and second period (Day 6–10) in *S*. *alterniflora* mesocosms, *K*. *obovata *mesocosms and their mixture, but had no effect in the third period (Day 11–15). This result suggests that discretely or infrequently measuring is inadequate to accurately predict the behavior of exotic N-induced response of N_2_O effluxes.

Meanwhile, with N addition, we found the mean N_2_O effluxes in the first period were higher than in the second period. We infered that, except for the higher N loading rates in the first period, the higher N_2_O effluxes may also be attributed to varying reponses of N_2_O effluxes to different N species. Previous study pointed out that N_2_O effluxes were larger when NH_4_^+^ -N was the dominant substrate and nitrification was the main source of nitrous oxide compared to the condition when nitrate and nitrite predominate [29]. In our study, major nitrogen species of input water were transformed from ammonium in the first period to nitrate in the second period with time, which could partly explain the reason for the different rates of N_2_O effluxes between the two periods.

Therefore, in order to get more accurate results especially in coastal wetlands which are frequently affected by tide, we should pay more attention to sampling time, daily N loading rates and N species of input water after N pulse addition. In addition, we added ammonium into seawater as the initial nitrogen species to assess the anthropogenic effects on N_2_O effluxes from coastal wetland, as it constitutes a major form of human inputs such as sewage and wastewater and sludge from aquaculture ponds. Future studies should also investigate the effects of other N species such as NO_3_^-^-N on N_2_O effluxes.

### Effects of plant invasion on N_2_O effluxes

Without N addition, there were no significant difference of N_2_O effluxes between the invasive *S*. *alterniflora* and native *K*. *obovata* mesocosms and more productive *S*. *alterniflora* did not result in higher N_2_O effluxes. This finding implied that the invasion of *S*. *alterniflora* played a limited role in mediating N_2_O effluxes from mangrove in N-limited condition. Previous research work have shown that fast-growing species such as *S*. *alterniflora*, a salt marsh species, increased N mineralization of soil to a greater extent than more conservative species such as *K*. *obovata*, a mangrove forest species [16, 45]. However, it has also been found that the rate of net N uptake was four- to six-fold higher for faster growing species than slower growing plant species (e.g., Poorter et al. (1991) [46]). Considering the high growth and high root uptake capacity of *S*. *alterniflora* [47], the mineralized N in soil could be largely consumed by *S*. *alterniflora*, which would result in no buildup of the soil mineral N pool. Therefore, without N addition, no difference in N_2_O effluxes between *S*. *alterniflora* and *K*. *obovata* mesocosms could be attributed to the similar concentration of soil N pool.

Meanwhile, we found significant antagonism effects of plant invasion and nitrogen loading on soil N_2_O effluxes. With N addition, the mean N_2_O effluxes from the invasive *S*. *alterniflora* were lower than that from native *K*. *obovata* mesocosms. In addition the increase rates of N_2_O effluxes by N addition were also lower in the *S*. *alterniflora* than native *K*. *obovata* mesocosms, indicating the invasion of *S*. *alterniflora* reduced N_2_O effluxes response to N loading in this simulated mangrove ecosystem. This result may be attributed to a crucial effect of vegetation type on the quality of N_2_O effluxes. On one hand, as important substrates participating in the processes of nitrification and denitrification by microbes [48], NH_4_^+^-N and NO_3_^-^-N would be absorbed by vegetation, which may inhibit N_2_O production due to the competition between the plant and microorganism for soil inorganic N. In our study, compared to mangrove mesocosms, higher biomass and higher uptake of N in *S*. *alterniflora* mesocosms were found, which might mean higher photosynthetic capacity, leading to stronger competition ability for N source and lower soil inorganic N content for N_2_O production by microbes. On the other hand, in wetland, plants with aerenchyma can supply oxygen to the rhizosphere [12, 13], which can form oxidized zone in the rhizosphere that stimulates nitrification and nitrification-denitrification processes [15, 49], therefore facilitating N_2_O effluxes. Previous researches have shown that stronger oxidation activity in the rhizosphere of mangrove species like *K*. *obovata* than salt marsh species such as *S*. *alterniflora* [50, 51]. Indeed, we found significant higher PNA in *K*. *obovata* mesocosms compared to *S*. *alterniflora* mesocosms. Therefore, we inferred that the higher N_2_O effluxes in the *K*. *obovata* mesocosms than in *S*. *alterniflora* mesocosms could be partly attributed to the stronger oxidation in the rhizosphere of mangrove species.

The mixture mesocosms of *S*. *alterniflora* and *K*. *obovata*, reflecting partly invasion of *S*. *alterniflora*, had higher plant richness compared to the monoculture *K*. *obovata* or *S*. *alterniflora*. With N addition, both partly and complete invasion (in monoculture *S*. *alterniflora* and mixture mesocosms) reduced N_2_O effluxes from mangrove soil in the first period. However, only complete invasion of *S*. *alterniflora* reduced N_2_O effluxes from mangrove soil in the second period. Moreover, both complete and partly invasion did not reduce N_2_O effluxes from mangrove soil with N addition in the third period and without N addition for all three periods. In conclusion, *S*. *alterniflora* invasion could reduce N_2_O effluxes regardless of invasion degree under relatively higher nitrogen availability, but no relief effect of *S*. *alterniflora* invasion on N_2_O effluxes occurred under N-limiting condition. The results indicated that N addition can improve the effect of partly *S*. *alterniflora* invasion on N_2_O effluxes in mangrove, perhaps because N greatly increased *S*. *alterniflora* growth and competition with mangrove seedlings [52], leading to exhibition of *S*. *alterniflora* characteristic in mixture mesocosms with N addition.

Although substrate condition and results of our controlled mesocosm experiment were similar to field experiment [27], cautions must be exercised when extrapolating results from controlled mesocosm studies to field-scale processes.

## Conclusions

N_2_O effluxes were significantly increased by the N loading in the *K*. *obovata*, the *S*. *alterniflora* and their mixture mesocosms, but the stimulatory effect of N loading on the N_2_O effluxes decreased gradually with time and dispeared when the N loading rates returned to the background level. The lower N_2_O effluxes and a weaker response to N addition in the *S*. *alterniflora* mesocosms than the *K*. *obovata* mesocosms were partly due to significantly higher growth of *S*. *alterniflora*, which could have led to lower increment of available N in the sediments for N_2_O production; and also due to higher oxidation capacity in the rhizosphere of *K*. *obovata*, which stimulated nitrification and nitrification-denitrification processes. Thus, *S*. *alterniflora* invasion into mangrove habitats could reduce N_2_O effluxes in case of amount of exogenous N loading, such as the input of sewage and wastewater and sludge from aquaculture ponds. These findings should be considered when projecting future N_2_O effluxes from global mangrove wetlands under the influences of both biological invasion and increasing exogenous N loading.

## Supporting Information

S1 Table Mass balance of nitrogen (g m^-2^) in the mangrove and *Spartina* mesocosms under different N loading during the experiment period from June 5, 2013 to July 5, 2013. (DOCX)
