# Supplementary material for: Co-Regulations of Spartina alterniflora Invasion and Exogenous Nitrogen Loading on Soil N2O Efflux in Subtropical Mangrove Mesocosms
Source: PLoS One. 2016 Jan 4;11(1):e0146199. doi: 10.1371/journal.pone.0146199 (PMC4701003; doi:10.1371/journal.pone.0146199)
Supplement: S1 Table — (DOCX) [file pone.0146199.s001.docx]

**Supporting information**

**S1 Table Mass balance of nitrogen (g m^-2^) in the mangrove and *Spartina* mesocosms under different N loading during the experiment period from June 5, 2013 to July 5, 2013.**

|  |  | **Total Input** | **Total Output** | **plant uptake** | **N_2_O-N** | **Sediment** | **Other loss** |
| --- | --- | --- | --- | --- | --- | --- | --- |
| N+ | KO | 149.6 | 100.8(67.4) | 9.7(6.5) | 0.7(0.5) | 2.9(1.9) | 35.5(23.7) |
|  | Mix | 149.6 | 102.4(68.5) | 36.5(24.4) | 0.5(0.3) | -13.1(-8.8) | 23.3(15.6) |
|  | SA | 149.6 | 103.9(69.5) | 43.5(29.1) | 0.3(0.2) | -20.2(13.5) | 22.1(14.8) |
| CK | KO | 4.8 | 2(41.1) | 4.6(96.5) | 0.1(2.2) | -2.5(-53.1) | 0.6(13.4) |
|  | Mix | 4.8 | 2(42.5) | 12.9(267.9) | 0.2(3.6) | -11.2(-232.3) | 0.9(18.4) |
|  | SA | 4.8 | 1.8(38.3) | 20.1(418.1) | 0.1(2.2) | -18(-374.7) | 0.8(16.1) |

a: plant uptake includes N accumulated in leaves, stems, propagules and roots; b: N_2_O-N is 2 times of the cumulative N_2_O-N emission during experiment period under the hypothesis that two water cycles in June shared the same cumulative N_2_O-N emission; c: Other loss involves N_2_ emission via nitrification–denitrification processes, ammonia volatilization; data in bracket show the percentages of total N inputs.
